# Relative Brain Perfusion Differences and Cognitive Improvement During Multimodal Management in a Patient With Progressive Cognitive Symptoms: A Case Report

**DOI:** 10.7759/cureus.116590

**Published:** 2026-09-20

**Authors:** Binh Pham

**Affiliations:** 1 Sleep Medicine, Amen Clinics, Costa Mesa, USA

**Keywords:** brain perfusion single-photon emission computed tomography (spect), cardiac arrest, case report, cerebrovascular disease, cognitive decline, cognitive impairment, cognitive screening, continuous performance test, functional neuroimaging, self-administered gerocognitive examination (sage)

## Abstract

Progressive cognitive symptoms in older adults may reflect multiple interacting neurologic, vascular, sleep-related, medical, and lifestyle factors, making both etiologic attribution and assessment of treatment response challenging. We report the case of a 72-year-old man with approximately five years of progressive cognitive symptoms, including short-term memory impairment, slowed processing, distractibility, reduced initiative, and executive difficulties, in the setting of a remote cardiac arrest, cardiovascular and cerebrovascular risk, subsequent head trauma, and suspected sleep-disordered breathing. Baseline evaluation included a Self-Administered Gerocognitive Examination (SAGE) score of 9/22, minimal depressive symptoms, continuous performance testing, and adjunctive resting and concentration brain perfusion single-photon emission computed tomography (SPECT). The SPECT findings were interpreted qualitatively as differences in relative regional tracer distribution and were not considered independently diagnostic. An individualized multimodal management plan emphasized sleep and circadian regularity, Mediterranean-style dietary measures, hydration, gradual aerobic and resistance exercise, cognitive and social engagement, and nonprescription nutritional supplementation without psychotropic medication. Over approximately six months, the patient and his wife reported improvement in memory, attention, organization, communication, motivation, and overall cognitive clarity, accompanied by renewed reading and cycling activity. Repeat SAGE increased by 9 points, from 9/22 to 18/22. Because several interventions were implemented concurrently, no single treatment component can be assigned a causal effect, and spontaneous improvement, natural variability, regression toward the mean, and practice effects cannot be excluded as contributors to the observed change. This case suggests that meaningful cognitive improvement may occur during individualized, multimodal longitudinal care while underscoring the importance of repeated reassessment and cautious interpretation of adjunctive brain perfusion SPECT.

## Introduction

Cognitive decline in older adults is often considered in the context of neurodegenerative disease, but its clinical course may also reflect multiple interacting contributors, including prior neurologic injury, cerebrovascular disease, sleep disruption, medical comorbidity, and potentially modifiable lifestyle factors. Evaluation is particularly challenging when several of these factors coexist, and no single etiology adequately explains the clinical presentation. Because a single assessment may capture only one point along a changing clinical trajectory, longitudinal observation, collateral history, standardized cognitive assessment, and consideration of potentially reversible contributors remain important alongside conventional diagnostic testing [1].

Acquired brain injury is an important consideration when interpreting later cognitive symptoms. Cognitive impairment is well described after cardiac arrest, with memory, attention, and executive functioning among the domains commonly affected in survivors [2]. Cerebrovascular disease may impose additional cognitive burden and frequently coexists with other age-related or acquired neurologic processes rather than acting as an isolated cause [3]. In older adults with a history of cardiac arrest, subsequent head trauma, cerebrovascular disease, and possible sleep-related contributors, the resulting cognitive phenotype may therefore represent a mixed clinical picture in which the relative contribution of each factor cannot be readily separated.

Potentially modifiable factors may further influence cognitive trajectory. Sleep-disordered breathing has been associated with cognitive impairment and cognitive decline in older adults [4]. Sleep quality, physical activity, vascular risk, and other medical or lifestyle factors may also affect cognitive performance. Improvement following intervention, however, does not establish the mechanism responsible for that change, particularly when several measures are introduced concurrently. Longitudinal reassessment is therefore important when evaluating whether observed changes reflect treatment-associated improvement, natural variability, or other factors.

Brain perfusion single-photon emission computed tomography (SPECT) is a functional nuclear medicine imaging technique that uses radiotracer distribution to provide a relative representation of regional cerebral perfusion. Although SPECT may provide adjunctive information in the evaluation of cognitive disorders, individual perfusion patterns lack sufficient specificity to establish the etiology of cognitive impairment in isolation [5]. Accordingly, SPECT findings should be interpreted in conjunction with the clinical history, cognitive assessment, and other available diagnostic information rather than as standalone evidence of a specific neurocognitive disorder. We report the case of a 72-year-old man with progressive cognitive symptoms in the setting of a remote cardiac arrest, subsequent head trauma, cerebrovascular disease, and possible sleep-disordered breathing who underwent adjunctive brain perfusion SPECT and individualized multimodal management. The clinical learning point is that meaningful cognitive improvement may occur during longitudinal, multimodal care even when the underlying etiology remains uncertain, while SPECT should remain an adjunctive rather than independently diagnostic component of the evaluation.

## Case presentation

A 72-year-old man presented on Day 1 for evaluation of gradually progressive cognitive symptoms that had developed over approximately five years and had become more noticeable during the preceding two years. His principal concerns involved short-term memory, cognitive processing, attention, organization, and task completion. He reported frequently repeating questions and having difficulty recalling recent conversations or events. His wife described increasing difficulty providing responses that were appropriately aligned with questions, often requiring repeated clarification before he arrived at the intended answer. She also noted difficulty accurately repeating information, with occasional paraphrasing that altered the intended meaning. Several close friends had independently commented on his increasing repetition of stories or information. Both the patient and his wife observed that stress appeared to worsen his cognitive performance.

Additional concerns involved executive functioning, motivation, and behavioral change. The patient described reduced initiative and follow-through, particularly for self-directed activities, despite remaining able to complete structured responsibilities. He reported longstanding difficulty with organization and time management but noted increasing distractibility, difficulty initiating tasks, and a tendency to become overwhelmed by lengthy to-do lists or remain focused on individual problems rather than moving on. His wife described decreased situational awareness while driving and a newer pattern of leaving cabinets, vehicle doors, or household entry doors open. He had also occasionally misplaced items such as his cell phone and, at times, did not immediately recognize familiar individuals after periods without contact. Long-term autobiographical memory was relatively preserved. His wife additionally noted reduced confidence and a less assertive interpersonal style than previously, together with somewhat more prolonged and intense social interactions.

His neurological and cardiovascular history was notable for a cardiac arrest approximately 4,000 days, or about 11 years, before Day 1 while cycling. According to the patient, he lost consciousness and was without spontaneous breathing or a detectable pulse for approximately three to four minutes before successful resuscitation. He subsequently required an eight-day medically induced coma. After regaining consciousness, he experienced prominent memory difficulties that gradually improved over the ensuing several months. He reported that his cardiologist had discussed the possibility that the cardiac arrest may have resulted in some degree of brain injury. Although his cognition improved substantially from the immediate post-arrest period, he and his wife later observed gradual recurrence and progression of cognitive difficulties over subsequent years. The original hospitalization, neurological evaluation, and neuroimaging records from the cardiac arrest were unavailable for retrospective review. Therefore, the presence, extent, and distribution of any hypoxic-ischemic brain injury could not be independently characterized.

Approximately 500 days, or about 1.5 years, before Day 1, he sustained a low-speed bicycle fall with impact to the right side of his head while wearing a helmet. He recalled being briefly dazed afterward but denied loss of consciousness. Several days later, he developed a subjective sensation of "lag" while walking and underwent neurological evaluation. Neuroimaging was reportedly interpreted as showing cerebrovascular narrowing involving the pontine region. Approximately 450 days before Day 1, he experienced a transient episode of delayed speech that was clinically diagnosed as a transient ischemic attack. According to the patient and his wife, subsequent vascular evaluation identified severe narrowing or near occlusion rather than complete obstruction. The original neuroimaging and vascular reports were unavailable for retrospective review. Accordingly, the involved vessel, degree of stenosis, associated parenchymal findings, and relationship of these events to his cognitive symptoms could not be independently characterized. His medical history was additionally notable for cardiovascular disease and prior myocardial infarction, with a family history of cardiovascular disease and stroke.

Sleep history was notable for intermittent difficulty initiating sleep and occasional nocturnal awakening to urinate, with variable difficulty returning to sleep. He generally obtained approximately 6.5-8 hours of sleep nightly and reported feeling refreshed upon awakening. His wife had previously observed episodes of apparent breathing pauses during sleep approximately one to two years before Day 1 and also reported occasional snoring. No formal sleep-study results were available for retrospective review. He additionally had a history of sleep bruxism identified by his dentist and had previously used a dental guard. He denied sleepwalking and did not report persistent daytime fatigue or other prominent sleep-related complaints at the initial evaluation.

Neuropsychiatric assessment further characterized difficulties with short-term memory, cognitive processing, attention, organization, and motivation. Depressive symptoms were minimal, with a Patient Health Questionnaire-9 (PHQ-9) score of 1 [6]. Brief cognitive screening with the Self-Administered Gerocognitive Examination (SAGE) yielded a score of 9/22, below the reported normal range [7]. A computerized continuous performance test was also administered [8]. Relative to the normative sample, response speed was slower; however, only one atypical T-score was identified, and the overall pattern was not interpreted as suggesting a disorder characterized by attention deficits. Overall, the presentation was characterized as a progressive multidomain cognitive syndrome involving memory, processing speed, executive functioning, attention, motivation, and behavior, with several plausible contributors including remote post-anoxic injury, cerebrovascular disease, possible sleep-disordered breathing, and an unexcluded neurodegenerative process.

The patient reported that routine blood testing had been performed approximately 90 days before Day 1 and that he had not been informed of clinically significant abnormalities. The original laboratory reports, individual analyte values, reference ranges, and scope of testing were unavailable for retrospective review. Accordingly, completion of commonly relevant studies such as vitamin B12, methylmalonic acid, thyroid-stimulating hormone, hemoglobin A1c, and lipid testing could not be verified, and the presence or absence of potentially reversible hematologic, metabolic, endocrine, nutritional, renal, or hepatic contributors to his cognitive symptoms could not be independently determined. No formal polysomnography or home sleep apnea testing results were available for retrospective review, and a comprehensive formal neuropsychological battery was not performed; cognitive assessment consisted of brief screening with the SAGE and computerized continuous performance testing. Review of prior testing and completion of additional studies, if clinically indicated, were therefore included in the initial management plan.

Following the Day 1 clinical evaluation, brain perfusion SPECT was obtained as an adjunctive functional imaging study using resting and concentration protocols. The clinical SPECT report documented use of technetium-99m hexamethylpropyleneamine oxime (99mTc-HMPAO), with the resting and concentration studies obtained on separate days.

During the concentration study, the patient performed the Conners Continuous Performance Test (CPT), a 15-minute computerized sustained-attention task [8]. According to the clinical SPECT report, radiotracer was administered three minutes after CPT performance began, after which the patient completed the task. The CPT served as a standardized sustained-attention condition during the concentration study rather than as a standalone diagnostic assessment of his cognitive symptoms. This protocol permitted descriptive comparison of relative regional tracer distribution during sustained attentional demand with that observed under resting conditions. The paired studies were interpreted descriptively and were not considered quantitative measurements of cerebral activation or absolute cerebral blood flow.

Technical acquisition information was abstracted from the clinical SPECT report rather than independently verified. The report described tomographic imaging approximately 30-60 minutes after radiotracer administration using high-resolution gamma-camera acquisition with fan-beam collimation, a 128 × 128 matrix, and 120 projections obtained at 3° intervals over 360°. Low-pass prefiltering and linear attenuation correction were applied, followed by reconstruction in coronal, sagittal, and transaxial planes and as three-dimensional surface renderings. The report documented use of a standardized linear display scale and surface maps depicting the most active 45% of brain activity. Patient-specific administered radiotracer activity, numerical scale values, validated quantitative regional thresholds, and complete acquisition and reconstruction metadata were unavailable for retrospective verification.

The reported regional increases and decreases were based on qualitative descriptions documented in the clinical SPECT interpretation rather than on validated quantitative regional analysis or a documented normative database comparison available for retrospective review. The resting study was described as having relatively decreased tracer activity involving the bilateral temporal lobes, inferior orbitofrontal cortex, prefrontal pole regions, bilateral occipital regions, and internal cerebellar regions. The temporal, prefrontal pole, and internal cerebellar decreases were described as more pronounced during the resting condition. The clinical interpretation additionally described relatively increased tracer activity involving the anterior cingulate, lateral prefrontal cortex, bilateral basal ganglia, a left focal thalamic region, middle cingulate, and posterior cingulate at rest. Representative resting surface-rendered brain perfusion SPECT images are shown in Figure 1.

**Figure 1:**
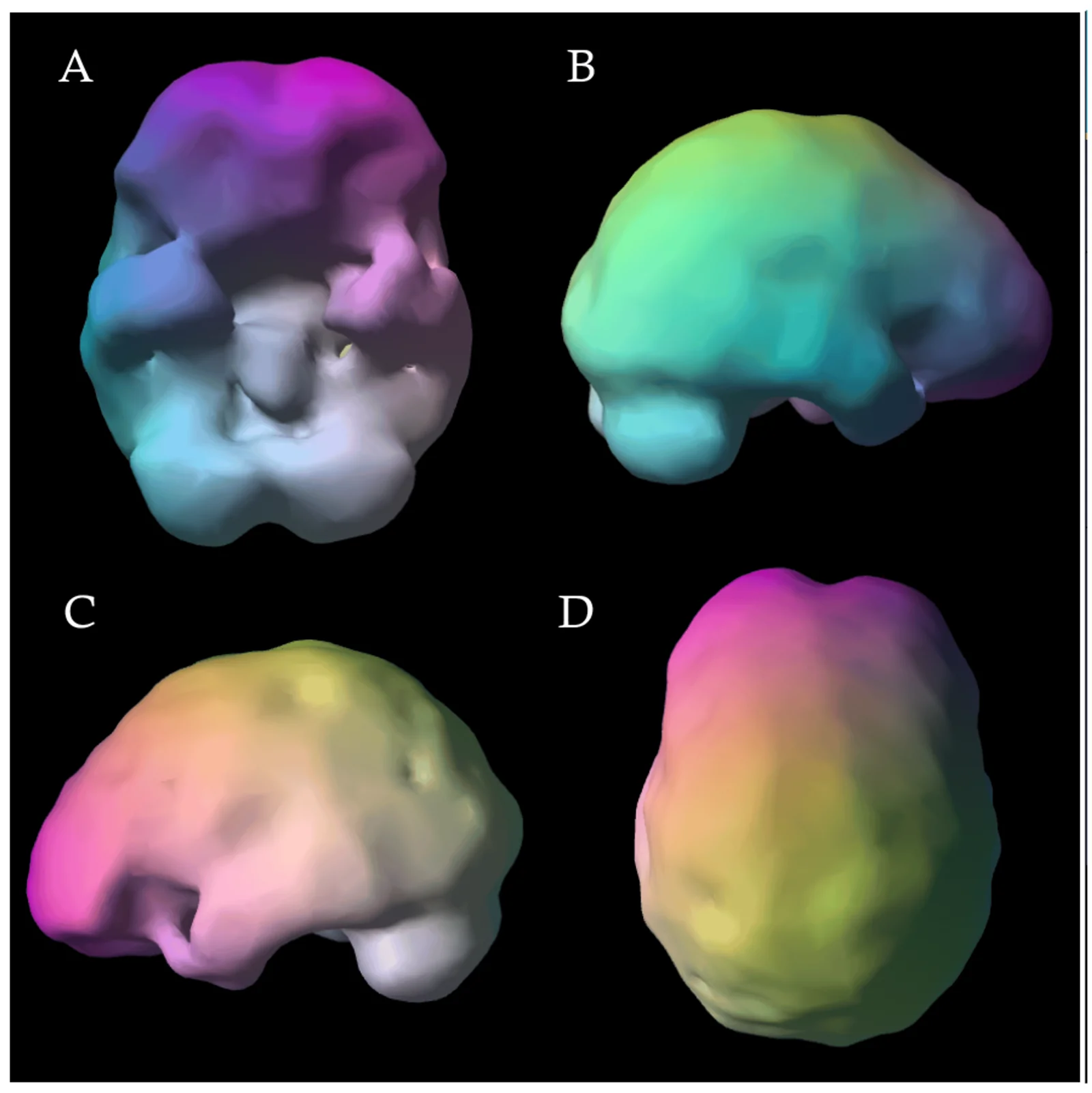
Resting surface-rendered brain perfusion SPECT images Resting surface-rendered brain perfusion single-photon emission computed tomography (SPECT) images showing relative regional tracer distribution. Warmer colors correspond to relatively greater displayed tracer activity, whereas cooler colors correspond to relatively lower displayed tracer activity within the reconstructed brain surface. (A) Inferior view showing the ventral cortical surface; (B) right lateral hemispheric view; (C) left lateral hemispheric view; (D) superior view showing the dorsal cortical surface. According to the qualitative interpretation documented in the clinical SPECT report, the resting study was described as having relatively decreased tracer activity involving the bilateral temporal lobes, inferior orbitofrontal cortex, prefrontal pole regions, bilateral occipital regions, and internal cerebellar regions. The clinical report documented use of a standardized linear display scale and surface maps depicting the most active 45% of brain activity; however, numerical scale values, an external reference distribution, and validated quantitative regional thresholds were unavailable for retrospective review. Accordingly, the displayed color and surface findings represent qualitative differences in relative tracer distribution and should not be interpreted as quantitative measurements of regional perfusion or absolute cerebral blood flow. The images are presented for descriptive and illustrative purposes and are not independently diagnostic.

The concentration study was described as having persistent relatively decreased tracer activity involving the bilateral temporal lobes, prefrontal pole regions, bilateral occipital regions, and internal cerebellar regions. According to the qualitative interpretation documented in the clinical SPECT report, the bilateral temporal, prefrontal pole, and internal cerebellar decreases were described as more pronounced during rest, whereas the bilateral occipital decreases were described as more pronounced during concentration. These comparisons were not based on validated quantitative between-session analysis. The clinical interpretation additionally described relatively increased tracer activity involving the anterior cingulate and lateral prefrontal cortex during both studies, with greater intensity during concentration. Increased right basal ganglia activity was described during both studies. Increased left basal ganglia activity was also described during both studies and was more intense during concentration. A medial focal thalamic increase was described during the concentration study. Increased middle and posterior cingulate activity was described during both studies, with the posterior cingulate finding described as more intense during concentration. These observations represented descriptive differences in relative regional tracer distribution and were interpreted in conjunction with the overall clinical evaluation rather than as independently diagnostic findings. Representative concentration surface-rendered brain perfusion SPECT images obtained during the CPT are shown in Figure 2.

**Figure 2:**
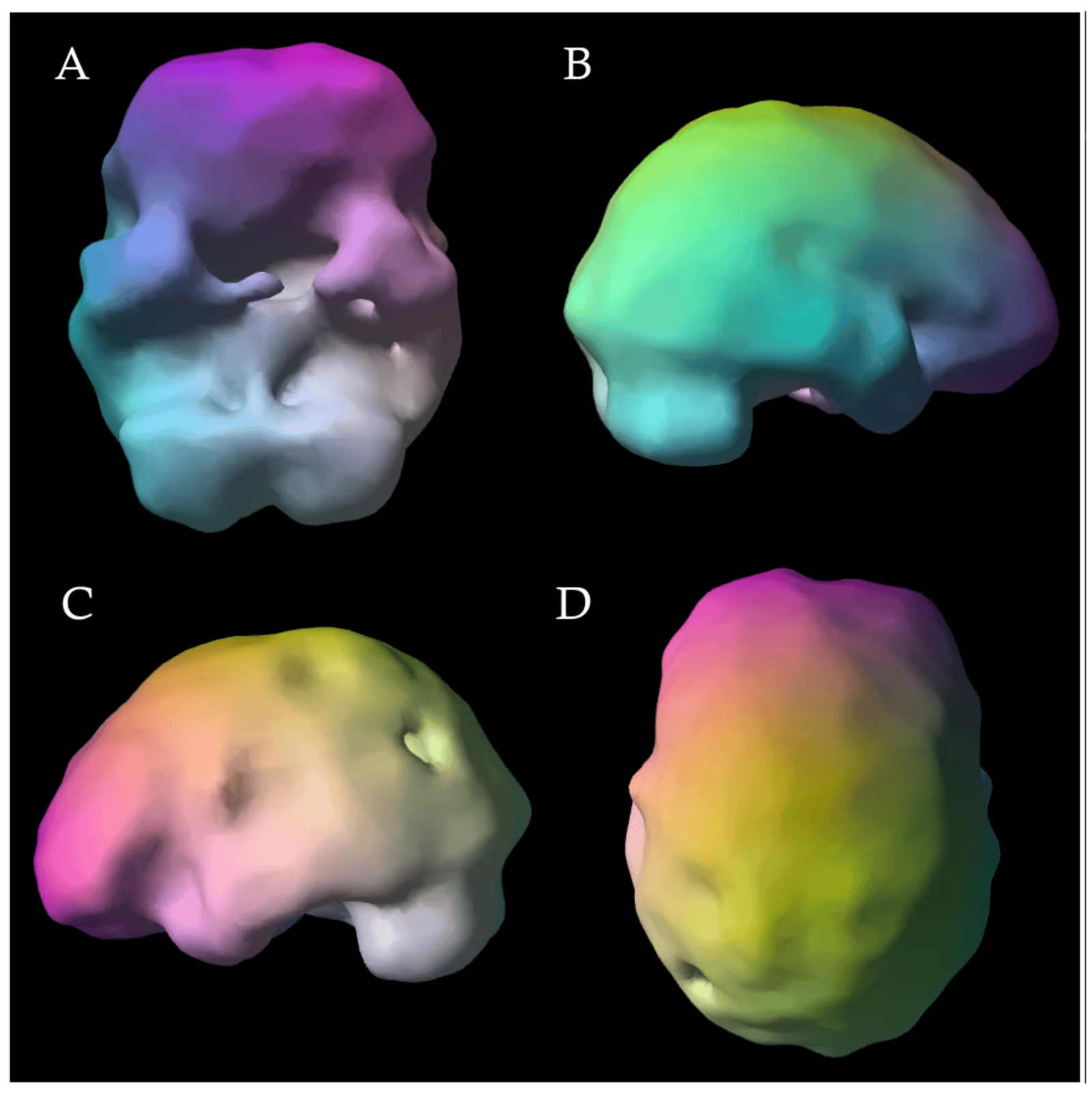
Concentration surface-rendered brain perfusion SPECT images obtained during the Conners Continuous Performance Test Concentration surface-rendered brain perfusion single-photon emission computed tomography (SPECT) images obtained during the Conners Continuous Performance Test (CPT), showing relative regional tracer distribution during sustained attentional demand. Warmer colors correspond to relatively greater displayed tracer activity, whereas cooler colors correspond to relatively lower displayed tracer activity. (A) Inferior view showing the ventral cortical surface; (B) right lateral hemispheric view; (C) left lateral hemispheric view; and (D) superior view showing the dorsal cortical surface. According to the qualitative interpretation documented in the clinical SPECT report, the concentration study was described as having relatively decreased tracer activity involving the bilateral temporal lobes, prefrontal pole regions, bilateral occipital regions, and internal cerebellar regions. The temporal, prefrontal pole, and internal cerebellar decreases were described as more pronounced during rest, whereas the occipital decreases were described as more pronounced during concentration; these comparisons were not based on validated quantitative between-session analysis. The clinical report documented use of a standardized linear display scale and surface-rendering methodology, but numerical scale values, an external reference distribution, and validated quantitative regional thresholds were unavailable for retrospective review. These findings should therefore not be interpreted as quantitative measurements of regional perfusion, cerebral activation, or absolute cerebral blood flow. Rest-concentration differences are presented descriptively, and the images are not independently diagnostic.

Following completion of the Day 1 evaluation, an individualized multimodal management plan was implemented. No psychotropic medication was initiated. Management focused on potentially modifiable contributors to cognitive and general health and included optimization of sleep and circadian regularity, adoption of a Mediterranean-style dietary pattern, adequate hydration, gradual incorporation of regular aerobic and resistance exercise, and maintenance of cognitive and social engagement. Nutritional supplementation included omega-3 fatty acids at a recommended dose of approximately 3,000 mg daily, daily vitamin D3 with vitamin K2, and a multinutrient cognitive-support powder titrated from one-half scoop twice daily to one scoop once or twice daily. Sleep recommendations emphasized consistent sleep and wake times, reduction of evening stimulation, and maintenance of a regular wind-down routine. Physical activity was to be increased gradually over the subsequent several weeks. Because these interventions were introduced concurrently and were not evaluated individually, no treatment effect could be attributed to any specific component of the management plan.

At approximately Day 60, the patient reported that his mind felt clearer and that his thinking and cognitive processing had become sharper and more organized. His wife independently described improved processing speed and communication, including less frequent interruption during conversation. A brief episode of anxiety related to concern about missing appointments was reported, although both the patient and his wife described improved awareness of his anxiety patterns overall. Mood was reported as good and improved, and sleep remained stable. He remained off psychotropic medication and continued the recommended lifestyle measures and supplement regimen. N-acetylcysteine (NAC) 1,200 mg twice daily was added at this visit after the initial cognitive and communication improvements had already been reported.

At approximately Day 180, the patient and his wife described further improvement in short- and long-term memory, attention, concentration, organization of thought, and overall cognitive clarity. He reported greater awareness of details during conversations and a more settled, less scattered thought process. Motivation had also improved, with renewed engagement in reading and cycling. He had rejoined his cycling club and completed a 30-mile ride. His wife independently provided similar observations regarding improvement in cognition and overall functioning. Mood remained stable without reported depression, anxiety, or irritability, and no sleep complaints or daytime fatigue were reported.

Repeat SAGE screening at approximately Day 180 yielded a score of 18/22 [7], representing a nine-point increase from the baseline score of 9/22 and placing the follow-up score within the reported normal range. The patient remained off psychotropic medications and continued the recommended lifestyle measures and nonprescription supplement regimen, including omega-3 fatty acids, vitamin D3 with vitamin K2, and NAC. The change in SAGE score was interpreted as improvement in brief cognitive screening performance rather than as evidence of reversal of a specific underlying neurocognitive disorder. Because several lifestyle and nutritional interventions were implemented concurrently and the clinical course was observed without a controlled treatment design, the relative contribution of any individual intervention to the observed cognitive and functional improvement could not be determined. The major clinical events, diagnostic assessments, interventions, and longitudinal outcomes are summarized in Table 1.

**Table 1:** Clinical timeline, diagnostic evaluation, interventions, and follow-up This table summarizes the documented clinical course, available diagnostic assessments, interventions, and longitudinal follow-up. Day 1 denotes the index evaluation. Relative timing of historical events before Day 1 is approximate because exact source dates were unavailable for retrospective verification. Original records from the prior cardiac arrest, outside neuroimaging and vascular evaluation, and reported laboratory testing were unavailable for retrospective review; corresponding findings are therefore presented as reported clinical history rather than independently verified results. Brain perfusion SPECT findings were interpreted descriptively and were not independently diagnostic. The observed longitudinal improvement occurred during a multimodal intervention, and no individual treatment component can be assigned a causal effect. CPT, Continuous Performance Test; HSAT, home sleep apnea testing; NAC, N-acetylcysteine; PHQ-9, Patient Health Questionnaire-9; PSG, polysomnography; SAGE, Self-Administered Gerocognitive Examination; SPECT, single-photon emission computed tomography

Clinical period	Clinical presentation	Evaluation and key findings	Management and outcome
Approximately 4,000 days, or about 11 years, before Day 1	Cardiac arrest while cycling with loss of consciousness and reported absence of spontaneous breathing or detectable pulse for approximately three to four minutes	Successful resuscitation followed by an eight-day medically induced coma. Prominent memory difficulties occurred after the event and gradually improved over several months. Original hospitalization, neurological, and neuroimaging records were unavailable for retrospective review	Substantial improvement from the immediate post-arrest cognitive impairment, followed over subsequent years by gradual recurrence and progression of cognitive symptoms
Approximately 500 days, or about 1.5 years, before Day 1	Bicycle-related head impact with brief dazed sensation and later subjective “lag” while walking	Neuroimaging was reportedly interpreted as showing cerebrovascular narrowing involving the pontine region. Original imaging and vascular reports were unavailable for review	Continued neurological and medical follow-up. The contribution of this event to the later cognitive course could not be independently characterized
Approximately 450 days before Day 1	Transient episode of delayed speech	The episode was clinically diagnosed as a transient ischemic attack, with subsequent vascular evaluation reportedly identifying severe narrowing or near occlusion. Original imaging and vascular reports were unavailable for review	Continued neurological and medical follow-up. The contribution of this event to the later cognitive course could not be independently characterized
Approximately 90 days before Day 1	No new cognitive event documented	The patient reported routine blood testing and had not been informed of clinically significant abnormalities. Original laboratory reports, analyte values, reference ranges, and scope of testing were unavailable for review. Completion of commonly relevant studies such as vitamin B12, methylmalonic acid, thyroid-stimulating hormone, hemoglobin A1c, and lipid testing could not be verified	Review of prior testing and additional laboratory evaluation, if clinically indicated, were included in the management plan
Day 1: Index evaluation	Approximately five years of progressive cognitive symptoms, increasingly noticeable over two years, including short-term memory impairment, repetition, slowed processing, executive difficulties, reduced initiative, distractibility, and collateral-reported behavioral and functional changes	SAGE 9/22; PHQ-9 1. CPT showed slower response speed with only one atypical T-score and was not interpreted as suggesting a disorder characterized by attention deficits. Sleep history included occasional snoring and previously observed breathing pauses. No formal PSG or HSAT results were available for retrospective review. A comprehensive formal neuropsychological battery was not performed. Resting and concentration brain perfusion SPECT were obtained on separate days, with the concentration study performed during the Conners CPT	No psychotropic medication initiated. Multimodal management emphasized sleep and circadian regularity, Mediterranean-style dietary measures, hydration, gradual aerobic and resistance exercise, cognitive and social engagement, omega-3 fatty acids at approximately 3,000 mg daily, daily vitamin D3 with vitamin K2, and a multinutrient cognitive-support powder titrated from one-half scoop twice daily to one scoop once or twice daily
Approximately Day 60	Clearer thinking with sharper and more organized cognitive processing	Wife independently reported improved processing speed and communication. Mood and sleep remained stable. Initial improvement was already evident before NAC was introduced	Continued multimodal lifestyle and nutritional regimen; NAC 1,200 mg twice daily was added after initial improvement had already been reported. No psychotropic medication was initiated
Approximately Day 180	Further improvement in short- and long-term memory, attention, concentration, organization of thought, motivation, and overall cognitive clarity	Wife independently corroborated cognitive and functional improvement. Repeat SAGE increased to 18/22. The patient resumed reading and cycling, rejoined his cycling club, and completed a 30-mile ride	Continued multimodal lifestyle and nutritional management without psychotropic medication. Improvement could not be attributed to any single component of the concurrent intervention plan

## Discussion

This case illustrates the difficulty of interpreting progressive cognitive symptoms when several potentially relevant neurologic, vascular, sleep-related, and lifestyle factors coexist. The patient experienced cognitive difficulties over several years in the setting of a remote cardiac arrest, subsequent traumatic and cerebrovascular events, and possible sleep-disordered breathing, yet no single factor adequately accounted for the entire clinical course. Longitudinal follow-up showed improvement across several domains, including subjective cognitive symptoms, collateral observations from his wife, functional engagement, and brief cognitive screening performance. This pattern does not establish a specific etiology for the preceding decline or a causal mechanism for the subsequent improvement. Rather, it highlights the value of reassessing cognitive trajectory over time and considering both historical and potentially modifiable contributors when the clinical presentation is multifactorial.

A remote cardiac arrest was one plausible contributor to the patient's later cognitive vulnerability. Cognitive impairment after cardiac arrest is well recognized, with memory, attention, and executive functioning among the domains commonly affected in survivors [2]. In this case, the patient experienced prominent memory impairment immediately after the 2015 arrest, followed by substantial improvement over the ensuing months. The later recurrence and gradual progression of cognitive symptoms therefore cannot be confidently attributed to the arrest alone. In addition, the original hospitalization records, neurological evaluations, and neuroimaging were unavailable for retrospective review, limiting assessment of whether hypoxic-ischemic injury occurred and, if present, its severity or distribution. The arrest is therefore best regarded as an important historical factor that may have contributed to cognitive vulnerability rather than as a definitive explanation for the subsequent clinical course.

Subsequent neurologic and cerebrovascular events introduced additional potential contributors to the patient's cognitive course. Cerebrovascular disease is an established contributor to cognitive impairment and may coexist with other age-related or acquired neurologic processes rather than acting as an isolated cause [3]. In this case, the 2024 bicycle-related head impact, subsequently reported cerebrovascular narrowing, and later transient ischemic attack added further complexity to interpretation of the progressive symptoms. However, the original neuroimaging and vascular studies were unavailable for retrospective review, preventing independent characterization of the involved vessel, degree of stenosis, associated structural findings, or relationship of these events to cognition. These later events therefore remain plausible contributors to the overall cognitive phenotype but cannot be assigned a definitive causal role.

Potentially modifiable contributors were also relevant to the clinical picture. The history of occasional snoring and previously observed breathing pauses raised concern for possible sleep-disordered breathing, which has been associated with cognitive impairment and cognitive decline in older adults [4]. More broadly, vascular risk, physical inactivity, and other lifestyle factors are increasingly recognized as potentially modifiable influences on cognitive health [9]. In this patient, the management plan addressed sleep regularity, physical activity, diet, hydration, and cognitive and social engagement rather than treating any single factor as an established cause of his symptoms. Because formal sleep-study results and the original laboratory data were unavailable for review, the extent to which sleep-related, metabolic, nutritional, or other reversible factors contributed to the presentation could not be determined. Nonetheless, identifying and addressing potentially modifiable contributors was clinically reasonable in the setting of persistent cognitive symptoms and diagnostic uncertainty.

The subsequent clinical improvement occurred during a multimodal intervention that included dietary modification, increased physical activity, sleep and circadian regularity, hydration, cognitive and social engagement, and nutritional supplementation. Multidomain lifestyle interventions have been associated with preservation or improvement of cognitive performance in at-risk older adults, providing a general rationale for this type of approach [10]. In this patient, improvement was supported not only by subjective report but also by collateral observations from his wife, improved communication and organization, resumption of reading and cycling, and an increase in SAGE score from 9/22 to 18/22 [7]. These findings support a meaningful longitudinal change in cognitive and functional performance but do not establish reversal of a specific neurocognitive disorder or identify which component of the management plan was responsible. Because several interventions were introduced concurrently and the clinical course was observed without a controlled design, causal attribution to any individual intervention is not possible. Spontaneous improvement, natural fluctuation in cognitive performance, regression toward the mean, and practice effects from repeat cognitive screening also cannot be excluded as contributors to the observed change.

Brain perfusion SPECT provided additional descriptive information but requires cautious interpretation. Although SPECT has been investigated as an adjunctive imaging modality in cognitive disorders, regional perfusion patterns may overlap across different conditions and generally lack sufficient specificity to establish etiology in isolation [5]. In this case, the reported regional increases and decreases reflected the qualitative clinical interpretation of relative 99mTc-HMPAO tracer distribution rather than validated quantitative regional measurements. The studies characterized relative regional tracer distribution associated with cerebral perfusion and were not acquired or analyzed as direct quantitative measurements of absolute cerebral blood flow [11]. Similarly, the apparent differences between the resting and concentration studies were descriptive and were not derived from validated quantitative between-session analysis.

The subsequent clinical improvement should not be interpreted as validating the baseline SPECT findings or demonstrating an imaging-defined treatment response. No repeat SPECT study was obtained, and there was no quantitative imaging endpoint against which the clinical course could be compared. Accordingly, the reported regional tracer patterns cannot establish that specific regions represented reversible dysfunction or that the multimodal intervention produced corresponding changes in cerebral perfusion. The imaging findings and longitudinal clinical improvement are therefore best regarded as parallel observations interpreted within the broader clinical context.

Several limitations should be acknowledged. This was a single-patient observational case without a controlled intervention, and multiple management strategies were implemented concurrently. Original records from the 2015 cardiac arrest, subsequent neuroimaging and vascular evaluations, reported laboratory testing, and formal sleep-study results were unavailable for retrospective review. Formal neuropsychological testing was not performed, and the SAGE is a brief cognitive screening instrument; therefore, practice effects and other sources of test-retest variability cannot be excluded when interpreting the increase from 9/22 to 18/22. The observational design also precludes distinguishing treatment-associated improvement from spontaneous recovery or natural variation over time. The SPECT studies lacked validated quantitative regional analysis, a documented normative comparator available for review, and formal inter-reader assessment. In addition, the resting and concentration studies were acquired on separate days and may have been influenced by physiologic variability. Despite these limitations, the convergence of patient-reported, collateral, functional, and screening-based improvement supports a meaningful longitudinal change in clinical status. The case underscores the value of continued reassessment and attention to potentially modifiable contributors when the etiology of progressive cognitive symptoms remains uncertain.

## Conclusions

This single case suggests that meaningful cognitive improvement may occur during individualized, multimodal longitudinal care even when the etiology of progressive cognitive symptoms remains uncertain. The observed improvement cannot be attributed to any single intervention, and spontaneous improvement or natural variability cannot be excluded. The main clinical lesson is the value of repeated reassessment and management of potentially modifiable contributors in patients with multifactorial cognitive decline. Brain perfusion SPECT may provide adjunctive descriptive information but should not be considered independently diagnostic or used to establish the mechanism of clinical improvement.
